# Associations Between Dementia Caregiver Network Complexity and Primary Caregiver Burden and Mental Health

**DOI:** 10.1093/geroni/igaf122.1169

**Published:** 2025-12-31

**Authors:** William McConnell, Katherine Haggar

**Affiliations:** Florida Atlantic University, Boca Raton, Florida, United States; Florida Atlantic University, Boca Raton, Florida, United States

## Abstract

Persons living with dementia (PLWD) are often supported by complex networks of interconnected informal and professional caregivers who perform a wide variety of caregiving-related tasks. Many primary caregivers are older adults themselves who have been thrown into a complicated and demanding care manager role with little training or support. We examine whether larger, more complex caregiver networks, including increased professional involvement, are a supporting resource or additional burden for primary caregivers at their center. We report results from a quantitative Social Network Analysis (SNA) study of 250 PLWD in the United States. SNA is a valuable tool for measuring complex networks without relying on reductive social interaction scales. We measured caregiver networks using SNA name generators to identify any contributors to ten caregiving-related tasks. Statistical analyses identified associations between caregiver network characteristics (size, composition, stability, supportiveness, and coordination) and primary caregiver well-being (PHQ-4 depression, PSS-4 stress, loneliness, and ZBI-4 burden). The sample included 131 primary caregivers age 60+ who were embedded in large networks of 9.1 secondary caregivers, on average (SD = 5.0, range=0-28). Larger caregiver networks were associated with higher primary caregiver stress (r=.25; p<.01) and burden (r=.26; p<.01). When primary caregivers had more frequent disagreements with others, they reported higher stress (r=.20; p<.05), loneliness (r=.18; p<.05), and burden (r=.29; p<.001). Professional composition and network stability were not associated with primary caregiver well-being, although more coordination was associated with perceived support and self-affirmation. Whereas others have highlighted the beneficial aspects of caregiver networks, we identify challenges in more complex caregiver systems.

